# Effects of ultrasound of different intensities plus neurotrophic factors on inflammatory markers and oxidative stress, and clinical efficacy in patients with diabetic peripheral neuropathic pain

**DOI:** 10.12669/pjms.41.9.11619

**Published:** 2025-09

**Authors:** Weili Ji, Meng Li, Lingzhi Li, Xue Bai, Fengjuan Wang

**Affiliations:** 1 Weili Ji Department of Pain, Affiliated Hospital of Hebei University, Baoding 071000, Hebei, China; 2 Meng Li Department of Pain, Affiliated Hospital of Hebei University, Baoding 071000, Hebei, China; 3 Lingzhi Li Department of Pain, Affiliated Hospital of Hebei University, Baoding 071000, Hebei, China; 4 Xue Bai Department of Pain, Affiliated Hospital of Hebei University, Baoding 071000, Hebei, China; 5 Fengjuan Wang Department of Pain, Affiliated Hospital of Hebei University, Baoding 071000, Hebei, China

**Keywords:** Ultrasound of different intensities, Neurotrophic factor, Diabetic peripheral neuropathic pain, Treatment

## Abstract

**Objective:**

To evaluate the efficacy of different ultrasound intensities combined with neurotrophic factors (NTFs) in diabetic peripheral neuropathic pain (DPNP) patients, focusing on pain relief, inflammation, and oxidative stress.

**Methodology:**

In this retrospective study, a total of 180 patients with DPNP admitted to Affiliated Hospital of Hebei University from October 2022 to December 2023 were randomized into control (gabapentin), low-dose (L: 0.5–1.0 W/cm²), and medium-dose (M: 1.0–1.75 W/cm²) ultrasound groups, all receiving 20 mg/day ganglioside for 5 weeks. Outcomes included Visual Analog Scale (VAS) scores, inflammatory markers (TNF-α, CRP, IL-6), and oxidative stress markers (SOD, GSH-Px, TAC, CAT, GR).

**Results:**

The L group showed higher overall response rates (88.33%) versus M (73.33%) and control (53.33%) (P=0.000). VAS scores decreased most in L group (P=0.00). TNF-α, CRP, and IL-6 levels reduced significantly in L/M groups versus control (P<0.05), with no intergroup differences (P>0.05). SOD, GSH-Px, TAC, and CAT levels improved most in L group (P=0.00). GR levels showed no significant difference among these groups (P = 0.88).

**Conclusion:**

Low-dose ultrasound combined with NTFs significantly alleviates DPNP pain, reduces inflammation, and enhances antioxidant capacity, demonstrating superior clinical efficacy.

## INTRODUCTION

With lifestyle changes in recent years, the incidence of diabetes and its complications has been increasing annually. Diabetic peripheral neuropathy (DPN) is the most common form of neuropathy caused by diabetes and a major causative factor of patient disability.^1^ The primary mechanism behind the development of neuritis in diabetic patients is hyperglycemia, which raises the osmotic pressure in the blood, leading to vascular wall damage and intimal injury, causing local blood flow disturbances and eventually neuropathy. Clinically, patients experience symptoms such as limb pain, numbness, and sensory loss, with severe cases progressing to foot ulcers, which significantly endanger the patient’s health and daily life.^2^

Traditional treatments for DPN mainly include blood sugar control, neurotrophic support, and drugs to improve microcirculation, with gabapentin being one of the most commonly used.^3^ The pathophysiology of diabetic peripheral neuropathic pain (DPNP) is complex, and its underlying mechanisms are not yet fully understood, which limits the effectiveness of single-treatment approaches. Research suggests that the best available evidence (Grade II evidence) exists for treating painful DPN. In particular, ultrasound therapy has proven to be effective in treating intractable pain and is an important non-pharmacological treatment option.^4^ NTFs play a crucial role in the growth, development, and maintenance of peripheral nerves, while gangliosides help maintain Na-K-ATPase activity on the surface of nerve cell membranes, promote nerve regeneration, and prevent the atrophy and degeneration of damaged nerve endings.^5^ This study aimed to explore the therapeutic effects of ultrasound of different intensities combined with NTFs on patients with DPNP, and some clinical progress has been achieved.

## METHODS

This was a retrospective study. A total of 180 patients with DPNP admitted to Affiliated Hospital of Hebei University from October 2022 to December 2023 were included and randomly divided into three groups: control group, low-dose ultrasound group (L group), and medium-dose ultrasound group (M group), with 60 patients in each group. Patients were randomized and stratified by baseline pain severity (VAS> 4) and diabetes duration to minimize confounding.

### Ethical approval:

The study was approved by the Institutional Ethics Committee of Affiliated Hospital of Hebei University (No.: HDFYLL-KY-2023-090; date: September 25, 2023), and written informed consent was obtained from all participants.

### Inclusion Criteria:

- Patients aged 35~75 years old.
- A confirmed diagnosis of Type-2 diabetes mellitus.^6^
- Meeting the diagnostic criteria for PNP.^7^
- Moderate or severe pain in the lower limbs or upper extremities (VAS score >4).
- Complete clinical and imaging data.
- Normal cognitive, mental, and language abilities, allowing accurate reporting of pain changes under the guidance of a physician.
- Good treatment compliance from both the patient and their family, with willingness and ability to cooperate in completing the study.
- No contraindications to the medications used in this study.
- No skin damage or injury in the affected area, making the patient eligible for ultrasound therapy;
- Signed informed consent.

### Exclusion Criteria:

- Patients in poor general condition with unstable vital signs.
- Patients with severe concomitant organic diseases such as liver or kidney dysfunction.
- Patients allergic to or intolerant of the medications involved in the study.
- Patients unable to cooperate with the study.
- Patients recently taking medications that could affect the study, such as immunosuppressants or hormones.

### Treatment methods:

Basic treatment included moderate physical activity, a low-salt, low-fat diet, and oral medication or insulin injections to control blood glucose levels. In addition to the basic treatment, the control group received standard treatment with gabapentin: Day one: 100 mg orally, three times a day (tid); Day two: 200 mg orally, tid; Day 3:300 mg orally, tid; maintenance treatment with 300 mg orally, tid for four weeks thereafter. Patients in the L group received low-intensity ultrasound and NTFs, specifically 20 mg of ganglioside, once daily, for five weeks, and ultrasound therapy with a dose of 0.5–1.0 w/cm². Patients in the M group received medium-intensity ultrasound and NTFs, including ganglioside administration as in the L group and ultrasound therapy with a dose of 1.0–1.75 w/cm². For ultrasound treatment, direct external scans were used, with iodinated glycerin as the coupling agent. The ultrasound probe was placed directly on the skin at the pain site and moved evenly in a spiral motion. The treatment duration depended on the affected area, with a maximum of 20 minutes per session, once daily, 10 sessions for a course, three courses in total.

### Outcome measures:

The same set of indicators were assessed in both groups after the end of treatment.

### Clinical efficacy evaluation:

Response to treatment was classified into four categories: complete response (CR): pain essentially disappeared, and sleep returned to normal; substantial response (SR): pain was significantly reduced, and sleep was occasionally disturbed by pain; partial response (PR): pain was somewhat alleviated, and sleep slightly improved; no response (NR): pain did not reduce, and sleep was affected. Overall response rate (ORR) = (Number of SR cases + Number of PR cases) / Total number of cases × 100%

### Pain assessment:

The self-reporting Visual Analog Scale (VAS) was used to assess pain levels in the three groups before and after treatment. The VAS score ranges from 0 to 10, where a lower score indicates less pain, and 0 represents no pain.^8^ Pain levels were assessed by patients independently, and the process was supervised by designated personnel.

### Inflammatory markers:

Peripheral venous blood samples (5 ml) were collected from all patients before and after treatment, and the levels of inflammatory markers, including tumor necrosis factor-alpha (TNF-α), C-reactive protein (CRP), and interleukin-6 (IL-6), were measured using enzyme-linked immunosorbent assay (ELISA).

### Oxidative stress markers:

Peripheral venous blood samples (5 ml) were taken before and after treatment. After separating the supernatant, oxidative stress markers were measured using ELISA, including superoxide dismutase (SOD), glutathione peroxidase (GSH-Px), total antioxidant capacity (TAC), catalase (CAT), and glutathione reductase (GR).

### Statistical analysis:

Statistical analysis was performed using SPSS 24.0. Measurement data were represented by (*X*±*S*). Continuous Variables (e.g., VAS scores, inflammatory/oxidative markers): Analyzed using one-way ANOVA for intergroup comparisons, followed by Tukey’s post-hoc tests. Paired t-tests were used for intragroup pre-post comparisons. Categorical Variables (e.g., treatment response rates): Assessed with Pearson’s *χ*² tests. A *p*-value of <0.05 was considered statistically significant.

## RESULTS

The L group included 27 males and 33 females, aged 57–74 years, with an average age of 65.05±5.71 years. The M group included 28 males and 32 females, aged 55–75 years, with an average age of 65.47±5.46 years. The control group included 25 males and 35 females, aged 55–73 years, with an average age of 64.75±5.80 years. There were no significant differences in general characteristics among the three groups, indicating comparability between groups (Table-I).

**Table-I:** Comparison of general patient information among the three groups (*X̄*±*S*, n = 60)

Item	L group	M group	Control group	F/χ^2^	P
Age (years)	65.05±5.71	65.47±5.46	64.75±5.80	0.242	0.785
Male (n, %)	27 (45.00%)	28 (46.67%)	25 (41.67%)	0.315	0.854
Body mass index (kg/m²)	34.65±3.76	34.59±4.25	34.87±3.70	0.084	0.919
***Affected body part***				0.323	0.851
Upper limb (n, %)	24 (40.00%)	26 (43.33%)	23 (38.33%)		
Lower Limb (n, %)	36 (60.00%)	34 (56.67%)	37 (61.67%)		
Disease course (years)	7.35±2.04	7.58±2.33	7.87±2.28	0.814	0.445
Tobacco consumption (n, %)	26 (43.33%)	28 (46.67%)	24 (40.00%)	0.543	0.762
Alcohol consumption (n, %)	18 (30.00%)	17 (28.33%)	21 (35.00%)	0.674	0.714
Hyperlipidemia (n, %)	24 (40.00%)	24 (40.00%)	30 (50.00%)	1.629	0.443

P > 0.05

The comparison of therapeutic effects among the three groups is shown in Table II. The results indicate that after treatment, the ORR was 88.33% in the L group, 73.33% in the M group, and 53.33% in the control group. The ORR in the L group was significantly higher than in the M group and the control group (*P* = 0.000).

**Table-II:** Comparison of clinical efficacy among the three groups (*X̄*±*S*, n = 60)

Group	SR	PR	NR	ORR*
L group	31	22	7	53 (88.33%)
M group	28	16	16	44 (73.33%)
Control group	18	14	28	32 (53.33%)
*χ* ^2^				18.222
P				0.000

* P < 0.05

There was no significant difference in the VAS scores among the three groups before treatment (*P* = 0.34). After treatment, the VAS scores decreased significantly compared to before treatment, with statistically significant differences (*P* = 0.00). The reduction in VAS scores in the L group was significantly greater than in the M group and the control group, with a statistically significant difference (*P* = 0.00) (Table-III).

**Table-III:** Comparison of VAS scores before and after treatment among the three groups (*X̄*±*S*, n = 60).

Group	Before treatment	After treatment*	t	P
L group*	7.42±1.31	4.07±0.90	33.382	0.000
M group*	7.62±1.18	5.13±0.79	16.028	0.000
Control group	7.53±1.29	6.22±1.21	8.400	0.000
F	0.380	71.835		
P	0.684	0.000		

* P < 0.05.

Before treatment, there were no significant differences in the serum level of TNF-α, CRP, or IL-6 among the three groups (all p > 0.05). After treatment, these markers decreased significantly compared to pre-treatment levels (TNF-α and IL-6, *P* = 0.00; CRP, *P* = 0.01). However, there was no significant difference between the L group and M group (TNF-α: *t* = 0.33, *P* = 0.74; CRP: *t* = 0.36, *P* = 0.72; IL-6: *t* = 1.24, *P* = 0.27) (Table-IV).

**Table-IV:** Comparison of serum inflammatory markers before and after treatment among the three groups (*X̄*±*S*, n = 60).

Marker	Time point	L group	M group	Control group	F	P
TNF-α (ng/L)	Before treatment	45.73±4.05	46.05±4.17	45.28±4.27	0.522	0.594
	After treatment*^Δ^	22.35±3.73	22.67±3.53	26.37±3.49	23.302	0.000
CRP (mg/L)	Before treatment	66.05±8.22	65.87±6.89	65.89±7.24	0.010	0.990
	After treatment*^Δ^	24.25±7.31	24.66±6.17	28.09±6.11	6.218	0.002
IL-6 (ng/L)	Before treatment	19.42±5.67	19.35±5.92	19.76±6.27	0.081	0.922
	After treatment*^Δ^	8.27±3.25	8.85±2.99	15.84±3.64	97.501	0.000

* P < 0.05 among the three groups;

Δ P > 0.05 between L group and M group.

After treatment, the levels of SOD, GSH-Px, TAC, and CAT were significantly higher in the L group than in the M group and control group (*P* = 0.00). There was no significant difference in GR levels among the three groups (*P* = 0.88) (Table-V).

**Table-V:** Comparison of serum antioxidant markers after treatment among the three groups (*X̄*±*S*, n = 60).

Group	SOD (U/mL)*	GSH-Px (U/mL)*	TAC (U/mL)*	CAT (U/mL)*	GR (U/mL)*
L group	84.73±6.36	368.57±18.35	18.27±2.66	14.05±2.48	126.84±13.23
M group	74.95±6.79	326.60±17.98	15.37±2.56	11.12±2.17	126.72±13.70
Control group	67.15±5.96	307.40±20.63	12.48±2.46	8.51±1.88	126.39±12.84
F	114.456	162.214	76.510	95.981	0.018
P	0.000	0.000	0.000	0.000	0.982

* P < 0.05

## DISCUSSION

Diabetic peripheral neuropathic pain (DPNP) is a common clinical type of neuropathic pain, and its treatment has always been a key and difficult point in clinical research.^9^ Based on a randomized controlled trial of 180 patients, this study systematically evaluated the clinical efficacy and mechanism of different intensity ultrasound combined with neurotrophic factors (NTFs) in patients with DPNP, and provided a new evidence-based basis for clinical treatment. The results showed that the overall response rate of the low-dose ultrasound (0.5-1.0 W/cm²) combined with ganglioside treatment group was significantly better than that of the medium-dose group and the control group (88.33% vs 73.33% vs 53.33%). This finding not only confirmed the superiority of the combination therapy, but also confirmed the superiority of the combination therapy. More importantly, the “dose window” effect of ultrasound therapy exists, that is, the higher the dose, the better the efficacy, which forms an important supplement to the previous understanding of ultrasound therapy.

In terms of mechanism discussion, this study provides a laboratory basis for the mechanism of combined treatment by detecting a series of inflammatory factors and oxidative stress indicators. After treatment, the serum levels of TNF-α, CRP and IL-6 in the low-dose group were significantly lower than those in the control group (P<0.01), and the antioxidant indexes such as SOD and GSH-Px were significantly increased (P<0.01). These findings echo Xia et al. ’s findings that low-intensity pulsed ultrasound (LIPUS) can improve the local microenvironment, but the present study further confirms that the improvement is more significant when LIPUS is combined with NTFs.^10^,^11^ Of particular note, there was no significant difference in GR levels among the three groups (P=0.88), a detail that suggests that different treatment modalities may have selective effects on oxidative stress pathways and provides important clues for subsequent mechanistic studies.

Compared with similar studies at home and abroad, the innovation of this study is mainly reflected in three aspects. First, based on the inflammatory mechanism theory proposed by Shillo et al.^12^, the positive correlation between anti-inflammatory effect and clinical efficacy was confirmed by clinical data. Secondly, it broke through the limitations of some researchs^13^-^16^ on the application of ultrasound alone, and systematically evaluated the combination regimen for the first time. Finally, the unique advantages of low-dose ultrasound were found, which provided a direct basis for the optimization of clinical operating parameters. These findings are of great value to improve the treatment system of DPNP.

From the perspective of clinical practice, the findings of this study have multiple implications. First, it provides a new option for patients who do not respond well to drug therapy or cannot tolerate the side effects of drugs. Second, the treatment is easy to operate, easy to promote in primary medical institutions; Third, the cost of treatment is relatively low, which helps to reduce the economic burden of patients. This combination therapy shows unique advantages, especially in elderly patients and those with long-standing poor glycemic control.^17^-^19^ At the basic research level, several phenomena identified in this study deserve further discussion. For example, why is low-dose ultrasound better? Is there any correlation between this and the cell cycle regulation theory proposed by Huang et al.?^20^ Does combination therapy have a selective effect on different types of nerve fibers? These questions need to be answered by more elaborate experimental design. Combined with recent research progress, we believe that the treatment of DPNP is developing towards multi-target and individualized direction. The value of this study is not only to confirm an effective therapy, but more importantly to show the broad prospects of physical therapy combined with drugs. Future studies can further explore the combination of ultrasound and other neurotrophic drugs, or try to extend this combination mode to other types of neuropathic pain treatment.

### Limitations:

Although the sample size was adequate for statistical analysis, the results may be more convincing if the sample is further expanded. The follow-up time is too short to evaluate the long-term efficacy. Without setting up a single ultrasound treatment group, it is difficult to fully distinguish the contribution of each factor in the combined treatment.

## CONCLUSION

Low-intensity ultrasound combined with neurotrophic factor has the advantages of definite efficacy, simple operation and good safety in the treatment of DPNP. This study not only provides a new treatment option for clinical practice, but more importantly, through systematic clinical observation and laboratory detection, the mechanism of action of the combination therapy is initially elucidated, which lays a solid foundation for subsequent research. With the deepening of research and accumulation of clinical experience, this combination therapy is expected to become an important part of the treatment system of DPNP.

### Future recommendations:

Multicenter studies with larger samples and longer follow-up periods are needed. It is worthwhile to design a control group of ultrasound treatment alone. Optimization of the treatment regimen with different interval cycles can be explored.

### Authors’ Contributions:

**WJ** and **ML:** Designed this study, prepared this manuscript, are responsible and accountable for the accuracy and integrity of the work. **LL** and **XB:** Data analysis, significantly revised this manuscript, analysis, and interpretation of data and draft the manuscript. **FW: Literature search**, Collected and analyzed clinical data. All authors have read and approved the final version of the manuscript.
